# Ontology-driven indexing of public datasets for translational bioinformatics

**DOI:** 10.1186/1471-2105-10-S2-S1

**Published:** 2009-02-05

**Authors:** Nigam H Shah, Clement Jonquet, Annie P Chiang, Atul J Butte, Rong Chen, Mark A Musen

**Affiliations:** 1 Centre for Biomedical Informatics, School of Medicine, Stanford University, Stanford, CA 94305, USA

## Abstract

The volume of publicly available genomic scale data is increasing. Genomic datasets in public repositories are annotated with free-text fields describing the pathological state of the studied sample. These annotations are not mapped to concepts in any ontology, making it difficult to integrate these datasets across repositories. We have previously developed methods to map text-annotations of tissue microarrays to concepts in the NCI thesaurus and SNOMED-CT.

In this work we generalize our methods to map text annotations of gene expression datasets to concepts in the UMLS. We demonstrate the utility of our methods by processing annotations of datasets in the Gene Expression Omnibus. We demonstrate that we enable ontology-based querying and integration of tissue and gene expression microarray data. We enable identification of datasets on specific diseases across both repositories. Our approach provides the basis for ontology-driven data integration for translational research on gene and protein expression data.

Based on this work we have built a prototype system for ontology based annotation and indexing of biomedical data. The system processes the text metadata of diverse resource elements such as gene expression data sets, descriptions of radiology images, clinical-trial reports, and PubMed article abstracts to annotate and index them with concepts from appropriate ontologies. The key functionality of this system is to enable users to locate biomedical data resources related to particular ontology concepts.

## Introduction and background

The amount and diversity of genomic scale data has been steadily increasing for the past several years. This increase has enabled integrative translational bioinformatics studies across these datasets [1]. Currently, the predominant genomic level data is gene expression microarrays. Recently, other forms of genomic scale measurements have been gaining acceptance, one of them being Tissue Microarrays. Tissue Microarrays allow for the immunohistochemical analysis of large numbers of tissue samples and are used for confirmation of microarray gene-expression results as well as for predictive pathology [2]. A single tissue microarray (TMA) paraffin block can contain as many as 500 different tumors, enabling the screening of thousands of tumor samples for protein expression using a few array sections [3]. Databases such as the Stanford Tissue Microarray Database (TMAD) [4] provide a central repository for data from TMA's akin to the Stanford Microarray Database (SMD) and Gene Expression Omnibus (GEO) for gene expression arrays.

Currently, it is difficult to integrate the results from gene expression microarrays and tissue microarrays data types and several reviews have suggested that it is essential to address this issue and synchronize the analysis, interpretation and data standards for these data [3,5]. In order to develop integrative approaches to interpret gene and protein expression datasets, there is a strong and pressing need to be able to identify all experiments that study a particular disease. A key query dimension for such integrative studies is the *sample*, along with a gene or protein name. As a result, besides queries that identify all genes that have a function X – which can be reliably answered using the Gene Ontology (GO) – we need to conduct queries that find all samples/experiments that study a particular disease and/or the effect of an experimental agent. However, because of the lack of a commonly used ontology or vocabulary to describe the diagnosis, disease studied or experimental agent applied in a given experimental dataset it is not possible to perform such a query.

The challenge is to create consistent terminology labels for each experimental dataset that would allow the identification of all samples that are of the same type at a given level of granularity. (e.g., *All carcinoma *samples versus *all Adenocarcinoma in situ of prostate *samples, where the former is at a coarser level of detail). One mechanism of achieving this objective is to map the text-annotations describing the diagnoses, pathological state and experimental agents applied to a particular sample to ontology terms allowing us to formulate refined or coarse search criteria [6,7]. Butte et al have previously applied a text-parser (GenoText) to determine the phenotypic and experimental context from text annotations of GEO experiments [1]. They report that text-parsing is still an inefficient method to extract value from these annotations [8]. In later work, Butte et al explored the use of PUBMED identifiers of the publication associated with GEO experiments and their assigned Medical Subject Headings (MeSH) to identify disease related experiments [8]. They were able to relate 35% of PUBMED associated GEO series to human diseases. Only half or so of the GEO experiments have PUBMED identifiers and the remaining are inaccessible to this approach, possibly necessitating alternative methods [8].

We have previously developed methods to process such text-annotations for tissue microarrays and map them to concepts in the NCI thesaurus and the SNOMED-CT ontologies [9,10]. In the current work we generalize our methods to process text annotations for gene expression datasets in GEO (as well as TMAD) and map them to concepts in the UMLS. We present results on the accuracy of our mapping effort and demonstrate how the mapping enables better query and integration of gene expression and protein expression data. We discuss the utility of our approach to derive integrative analyses. We believe that a similar integration problem exists for other kinds of data sources: for example, a researcher studying the allelic variations in a gene would want to know all the pathways that are affected by that gene, the drugs whose effects could be modulated by the allelic variations in the gene, and any disease that could be caused by the gene, and the clinical trials that have studied drugs or diseases related to that gene. The knowledge needed to study such questions is available in public biomedical resources; the problem is finding that information.

Currently most publicly available biomedical data are annotated with unstructured text and rarely described with ontology concepts available in the domains. The challenge is to create consistent terminology labels for each element in the public resources that would allow the identification of all elements that relate to the same type at a given level of granularity. These resource elements range from experimental data sets in repositories, to records of disease associations of gene products in mutation databases, to entries of clinical-trial descriptions, to published papers, and so on. Creating ontology-based annotations from the textual metadata of the resource elements will enable end users to formulate flexible searches for a wider range biomedical data besides just gene expression arrays and tissue arrays [6,7,9,11]. Therefore, the key challenge is to automatically and consistently annotate the biomedical data resource elements to identify the biomedical concepts to which they relate. Expanding on our preliminary work with GEO datasets and tissue microarray annotations, we have built a prototype system for ontology based annotation and indexing of biomedical data in the BioPortal ontology repository [12]. The system's indexing workflow processes the text metadata of several biomedical resource elements to annotate (or tag) them with concepts from appropriate ontologies and create an index to access these elements. The key functionality of this system is to enable users to find biomedical data resources related to particular ontology concepts.

## Methods

### Overview of annotations in GEO

The Gene Expression Omnibus (GEO) is an international repository of microarray data run by the National Center for Biotechnology Information (NCBI). In this work we use the November 2006 release of GEO, which contained 108371 samples, 4593 GEO experiment series and 1080 GEO datasets, 369 of which are human. In this analysis we focus only on the human datasets. Each GEO dataset has a title and a description field that contain text entered by the person uploading the dataset. Moreover, GEO datasets can have an additional 24 descriptors (such as agent, cell line, and species) along with their subset descriptions. In the current work we process the text from the title, description and agent descriptors of GEO datasets.

### Overview of text annotations in TMAD

The Stanford Tissue Microarray Database (TMAD) contains data from immunohistochemical analyses performed with tissue microarrays. The TMAD provides tools for quick upload, storage and retrieval of the tissue microarray images and the analysis of immunohistochemical staining results [13]. Each sample in the TMAD contains free-text annotations – entered by the experimenter – for fields such as the organ system, and up to five diagnosis terms (one principal diagnosis field and four sub diagnosis fields) describing the sample. We concatenate the text from these six fields and use it as the annotation of the sample for our work. We refer to experiments studying samples with the same diagnoses as one dataset. Currently, the TMAD has 10734 samples that can be grouped into 1045 datasets according to their diagnoses.

### Indexing with ontology terms

We downloaded UMLS 2006 AD and created a MySQL database using the Metamorphosys tool as described in the UMLS documentation [14].

In order to map existing annotations in TMAD and GEO to ontology terms, we used the UMLS-Query module developed by our group to process the existing descriptions of the samples and matching them to ontology terms. Fully describing the UMLS-Query module and all of its functionality is beyond the scope of this current work but we describe the key mapping function *mapToId *here. For each text-annotation, we read a sliding window of five words from the text. We generate all possible permutations (5-grams) of these words and look for an exact match to an ontology term. We examine all 5 word permutations because we observed that most disease and drug names are less than 5 words in length. This permutation based method, though accurate, can be made more efficient computationally. We are working on that collaboratively with the National Center for Integrative Biomedical Informatics [15]. We restrict the matches to SNOMEDCT and the NCI thesaurus vocabularies when identifying disease names. The UMLS-Query module along with detailed documentation is available from [16]. We do not employ any natural language processing strategies such as stemming, normalization or noun-phrase recognition. We also do not employ any heuristics or hacks for increasing match accuracy.

The result of the mapping is a table that associates each GEO or TMAD dataset identifier to one or more concepts in the UMLS. We query this table to identify disease related datasets as well as identify matching datasets from the repositories.

### Use of mgrep

For our prototype we used a tool called mgrep [15], developed by University of Michigan. Mgrep implements a novel radix tree based data-structure that enables fast and efficient matching of text against a set of ontology terms. We use mgrep instead of UMLS-Query in our prototype because of mgrep is several times faster than UMLS-Query and the key idea implemented in the *mapToId *function of UMLS-Query is also implemented in mgrep.

### Architecture of the prototype system

**Figure 1:**
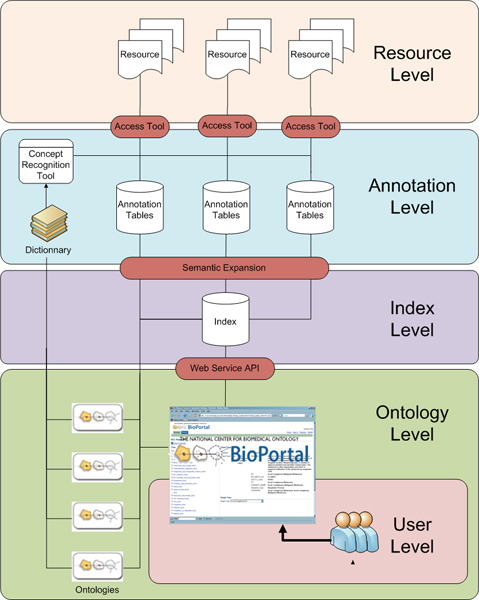
**Architecture of our prototype system comprising of different levels**. The figure shows the architecture of the prototype consisting of different levels (0). The *resource level*, provides and abstraction for elements (*E1...En*) of public biomedical resources (such as GEO and PubMed). The *annotation level*, uses a concept recognition tool called mgrep (developed by Univ. of Michigan) to annotate (or tag) resource elements with terms from a dictionary; constructed by including all the concept names and synonyms from a set of ontologies accessible to the *ontology level*. The annotation tables contain information of the form "*element E was annotated with concept T in context C*". At the *index level*, a global index combines all the annotations to index them by ontology concepts and provides information for the form "*Concept T annotates elements E1, E2,..., En*". See main text for details.

In this section we describe the architecture of the prototype consisting of different levels as shown in Figure 1. The *resource level*, provides and abstraction for elements of public biomedical resources (such as GEO and PubMed). An element is identifiable and can be linked by a specific URL/URI (id), and it has a structure that defines the metadata contexts for the element (title, description, abstract, and so on). We retrieve the element's text metadata from resources using public APIs, and keep track of the original metadata and element id. The *annotation level*, uses a concept recognition tool called mgrep (developed by Univ. of Michigan) to annotate (or tag) resource elements with terms from a dictionary. The dictionary is constructed by including all the concept names and synonyms from a set of ontologies accessible to the ontology level. The annotation process keeps track of the context (such as title, description) from which the annotation was derived. The results are stored as annotation tables, which contain information of the form "*element E was annotated with concept T in context C*". At the *index level*, a global index combines all the annotation tables to index them by ontology concepts. The index contains information for the form "*Concept T annotates elements E1, E2,..., En*".

We use relations contained in the source ontologies to expand the annotations. For example, using the is_a relation, for each term we create additional annotations according to the parent-child relationships of the original concept; a process we refer to as the transitive closure of the annotations. As a result, if a resource element such as a GEO expression study is annotated with the concept *pheochromocytoma *from the National Cancer Institute Thesaurus (NCIT), then a researcher querying for *retroperitoneal neoplasms *can find data sets related to *pheochromocytoma*. The NCIT provides the knowledge that *pheochromocytoma *is_a *retroperitoneal neoplasms*. Creation of annotations based on transitive closure step is done offline because, processing the transitive closure is very time consuming – even if we use a pre-computed hierarchy – and will result in prolonged response times for the users. This use case is similar, in principle, to query expansion done by search engine like Entrez; however, Entrez does not use the NCIT, therefore, even though *pheochromocytoma *related GEO data sets exist, none show up on searching for retroperitoneal neoplasms in Entrez. In our system, however, a researcher could search for *retroperitoneal neoplasms *and find the relevant samples.

At the *user level*, on searching for a specific ontology concept, the results provide resource elements annotated directly or via the step of transitive closure. The user receives the result in terms of references and links (URL/URI) to the original resource elements. The system architecture illustrates the generalizability of our implementation. Note the same model could be applied for domains other than biomedical informatics. The only specific components of the system are the resource access tools (which are customized for each resource) and, of course, the ontologies.

### Example demonstrating the processing of a GEO dataset in the prototype system

A GEO dataset represents a collection of biologically – and statistically – comparable samples processed using the same platform. We treat each GEO dataset as a resource element and process its metadata. Each GEO dataset has a title and a summary context that contain free text metadata entered by the person creating the dataset. For example the GEO dataset 'GDS1989', which is available online [17] and can be retrieved using the EUtils API [18], has the title *Melanoma progression*. GDS1989's summary contains the phrase: *melanoma in situ*. The Human disease ontology[19], provides the concept *Melanoma *in our system's dictionary. Therefore, our concept recognition tool produces the following annotations:

*Element GDS1989 annotated with concept DOID:1909 in context title*;

*Element GDS1989 annotated with concept DOID:1909 in context summary*;

The structure of the Human disease ontology shows that DOID:1909 has 36 direct or indirect parents such as for instance DOID:169, *Neuroendocrine Tumors *and DOID:4, *Disease*, therefore the transitive closure on the is_a relation generates the following annotations:

*Element GDS1989 annotated with concept DOID:169 with closure*;

*Element GDS1989 annotated with concept DOID:4 with closure*;

## Results

We processed the annotations corresponding to the annotations of 369 GEO datasets (GDS) and 1045 TMAD datasets available at the time this work was conducted. We then evaluated the ability of our ontology-based indexing scheme to enable the identification of experiments for the following use cases: 1) Accurately identify experiments related to particular diseases 2) Identify gene and protein expression datasets corresponding to diseases from both GEO and TMAD. These use cases were defined based on current research in translational bioinformatics and prior reviews indicating the need for such integrative analyses [1-3,8].

### Identifying disease related experiments in GEO

Out of 369 GDS, we identify 241 disease related experiments. The 241 disease related experiments can be grouped into categories according to the semantic type of the concepts assigned to them as shown in Table 1. We exclude high level ontology terms such as *cancer, syndrome, exposure, damage, toxicity *before performing the grouping. The number of experiments identified as disease related drops to 209 on removing high level terms. Such terms, though accurately mapped and being of the right semantic type, are too high level to enable the correct identification of disease related datasets.

**Table 1:** Categorization of GEO datasets according to the Semantic type after excluding matches to high level ontology terms.

Semantic type	Number of GDS
Neoplastic Process	109
Disease or Syndrome	97
Injury or Poisoning	8
Mental or Behavioral Dysfunction	3

Table 2 shows examples of the highest and lowest numbers of GEO datasets for the *Neoplastic Process *and *Disease or Syndrome *categories. (We omit rows that subsume other rows, for example leukemia subsumes acute leukemia, which in turn subsumes acute myeloid leukemia)

**Table 2:** Overview of the number of GEO datasets for concepts in the *neoplastic process *and *disease or syndrome *category

GDS	Concept name	CUI	Semantic type
Examples of cancers with many GEO datasets			

26	Breast cancer	C0006142	Neoplastic Process
11	Acute myeloid leukemia	C0023467	Neoplastic Process
5	Acute lymphoblastic leukemia	C0023449	Neoplastic Process

Examples of cancers with few GEO datasets			

1	Kaposi's sarcoma	C0036220	Neoplastic Process
1	Acute promyelocytic leukemia	C0023487	Neoplastic Process
1	Pleural mesothelioma	C1377913	Neoplastic Process

Examples of diseases with many GEO datasets			

13	Duchenne dystrophy	C0013264	Disease or Syndrome
6	Arthritis	C0003864	Disease or Syndrome
4	Chronic obstructive pulmonary disease	C0024117	Disease or Syndrome

Examples of diseases with few GEO datasets			

1	Open-angle glaucoma	C0017612	Disease or Syndrome
1	Purpura thrombocytopenic	C0857305	Disease or Syndrome
1	Corneal dystrophy	C0010035	Disease or Syndrome

### Identifying disease related experiments in TMAD

We have previously presented results on processing annotations in TMAD. For the current work, we reprocessed the records in TMAD and preformed the evaluation as described before [9]. The average precision and recall was 85% and 95% respectively. The annotations of 1045 datasets mapped to 902 disease related concepts in the UMLS. We do not discuss this further because this has been described in our previous work [9].

### Identifying matching GEO and tissue array datasets

From the 902 disease related datasets in TMAD and 241 disease related GDS that we identified in GEO, we were able to identify 45 disease related concepts for which there were datasets in both GEO and TMAD – and hence are potential candidates to support further analysis. Many of them are high level matches (such as Leukemia) that are accurate but too high level to enable correlative analyses.

From this set of 45 matches, we identified the 23 disease related concepts that were at an appropriate level of granularity and have multiple samples in both GEO and TMAD to enable further integrative study. In Table 3 we show the number of datasets, the number of GEO samples (GSM) and the corresponding number of tissue microarray samples for these.

**Table 3:** Diseases for which there are both gene expression and tissue microarray datasets

Disease	GEO datasets	GEO samples	TMAD samples
Acute myeloid leukemia	11	366	3
Malignant melanoma	3	47	43
B-cell lymphoma	3	133	27
Prostate cancer	3	47	15
Renal carcinoma	2	34	185
Carcinoma squamous	2	105	175
Multiple myeloma	2	225	169
Clear cell carcinoma	2	34	63
Renal cell carcinoma	2	34	9
Breast carcinoma	2	3	1277
Hepatocellular carcinoma	1	80	163
Carcinoma lung	1	91	66
Cutaneous malignant melanoma	1	38	41
T-cell lymphoma	1	29	31
Lymphoblastic lymphoma	1	29	30
Uterine fibroid	1	10	19
Medulloblastoma	1	46	9
Clear cell sarcoma	1	35	8
Leiomyosarcoma	1	24	5
Mesothelioma	1	54	5
Kaposi's sarcoma	1	4	3
Cardiomyopathy	1	14	2
Dilated cardiomyopathy	1	14	2

### Evaluation

In order to calculate the precision and recall for the task of identifying disease related experiments in GEO, we examined the 241 (209 after removing high level terms) GDS identified as disease related, to determine which of them were correctly identified. One of the authors (APC), went through each matched record and scored them for being a true positive of being a false positive. For calculating recall, we examined the unmatched GDSs (from the total of 369 GDSs) and searched the UMLS manually to identify concept to which the GDSs should have matched; this allowed us to compute the false negative rate. (Note that such exhaustive evaluation is not possible with the current size of GEO, which has over 2000 GDSs). The results of this evaluation are presented Table 4.

**Table 4:** Accuracy of identifying disease related datasets

Accuracy in identifying disease related datasets			
	Correct	Incorrect	Total

Positive	202 (TP)	39 (FP)	241
Negative	97 (TN)	31 (FN)	128
Precision = 83.8%		Recall = 86.6%	

Accuracy in identifying disease related datasets after limiting high level matches			

	Correct	Incorrect	Total

Positive	188 (TP)	21 (FP)	209
Negative	115 (TN)	45 (FN)	160
Precision = 89.9%		Recall = 80.6%	

Next, we evaluate the ability to accurately match up the right tissue array datasets with gene expression datasets. Out of the 45 candidate datasets proposed as corresponding between GEO and TMAD, on manual inspection all of them were accurate matches, though 12 were high level terms such as *Cancer, Syndrome, and Sarcoma*. We consider these as false positive because such matched are uninformative for the purpose of matching up disease related datasets across repositories. This gives us a precision of 73%. We were unable to perform a recall analysis for this task because it is extremely time consuming to manually examine all the TMAD and GEO datasets to determine the number of matches that were missed by our method. One notable disease for which datasets exist in both but were not accurately identified is Breast carcinoma. Most datasets in GEO for this disease are labeled with the term Breast Cancer (C0006142) and those in TMAD are labeled with the term Breast carcinoma (C0678222). These terms have different CUIs in the UMLS and hence we were unable to match up these datasets.

### Integration of the prototype system with NCBO BioPortal

The National Center for Biomedical Ontology (NCBO) [11] develops and maintains a Web application called BioPortal to access biomedical ontologies. This library contains a large collection of ontologies, such as GO, NCIT, International Classification of Diseases (ICD), in different formats (OBO, OWL, etc.). Users can browse and search this repository of ontologies both online and via a Web services API.

**Figure 2:**
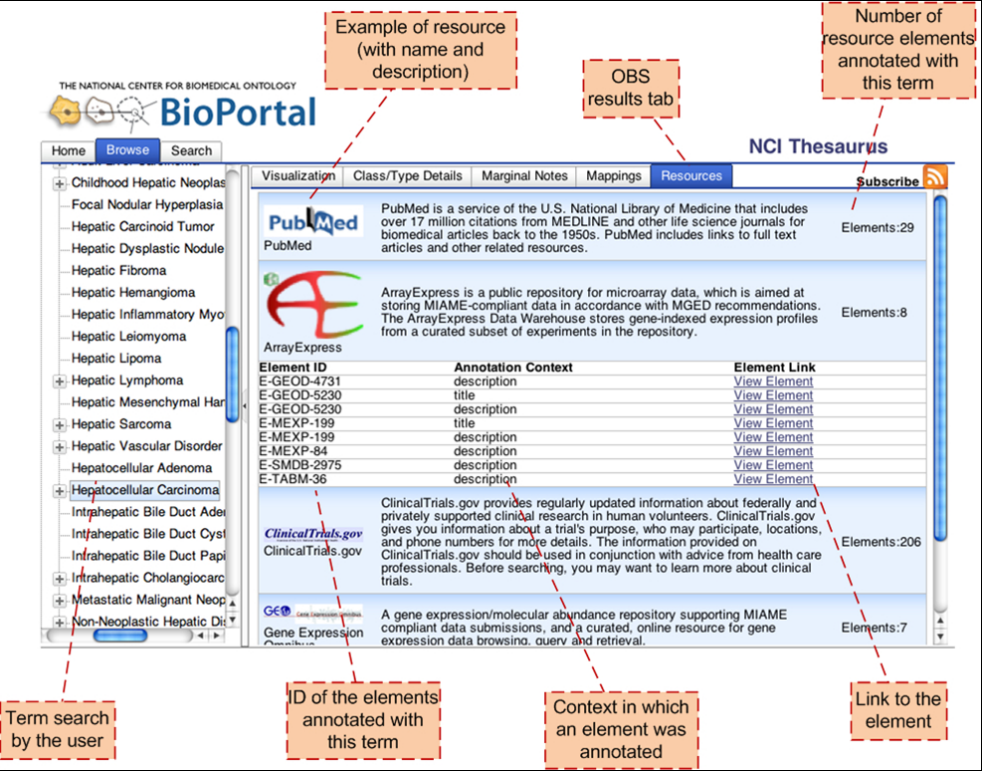
**User interface within BioPortal**. The figure shows the view seen by a user browsing the NCIT in BioPortal and selecting an ontology concept (in this case, *Hepatocellular carcinoma*). The user can see the numbers of online resource elements that relate directly to that concept (and the concepts that it subsumes). The interface allows the user to directly access the original elements that are associated with *Hepatocellular carcinoma *for each of the indexed resources.

We implemented the first prototype of the system as in the methods section. We have written a set of Java access tools to access five resource databases. The resources processed and the numbers of annotations currently available in our system are presented in Table 5. A public representational state transfer (REST) services API [12] is available to query the annotations and we have used this API to integrate the system with BioPortal as shown in Figure 2.

**Table 5:** Number of elements annotated from each resource in the current prototype

**Resource**	Number of elements	Resource local size (Mb)	Number of direct annotations (mgrep results)	Total number of 'useful'^1 ^annotations	Average number of annotating concepts
**PubMed (subset)**	1050000	146.1	30822190	174840027	763
**ArrayExpress**	3371	3.6	502122	1849224	525
**ClinicalTrials.gov**	50303	99	16108580	48796501	824
**Gene Expression Omnibus**	2085	0.7	165539	772608	359
**ARRS GoldMiner (subset)**	1155	0.5	134229	662687	564
**TOTAL**	1106914	249.9	47732660	226921047	(avg)461.5

^1 ^We do not add a closure annotation between an element and a concept in the index if the given element was already directly annotated with the given concept.

In our prototype, we have processed: (1) gene-expression data sets from GEO and Array Express, (2) clinical-trial descriptions from Clinicaltrials.gov, (3) captions of images from subset of ARRS Goldminer, and (4) abstracts of a subset of articles from PubMed. Table 5 shows both the current number of elements annotated and the number of annotations created from each resource that we have processed. Our prototype uses 48 different biomedical ontologies that give us 793681 unique concepts and 2130700 terms. As a result of using such a large number of terms, our system provides annotations for almost 100% of the processed resources. The average number of annotating concepts is between 359 and 769 per element, with an average of 27% of these annotations resulting from directly recognizing a term. In the current prototype, concept recognition is done using a tool developed by National Center for Integrative Biomedical Informatics (NCIBI) called *mgrep *[20]. We have conducted a thorough analysis of the accuracy as well as scalability and flexibility of mgrep in recognizing concepts representing disease names, body parts and biological processes. The prototype design of the annotation level is such that we can plug-in other concept recognizers. The prototype is available online at [12].

## Discussion and future work

In the current work we have processed annotations of tissue microarray samples in TMAD as well as annotations of gene expression datasets in GEO and mapped them to concepts in the UMLS. One of the insights in our work is that disease names are rarely longer than five words and it is computationally tractable to perform an exhaustive search for all possible five-word permutations of the text-annotation.

Mapping text-phrases to UMLS concepts has been performed by other researchers in the past [21,22]. Most of these approaches are for the purpose of automatic indexing of biomedical literature, and have been shown to be inadequate for processing annotations of high-throughput datasets [1,8]. It has also been shown that for the task of identifying concepts from annotations of high-throughput datasets, simple methods perform equal or better than Metamap [1,8,10,15]

In the current work, we use very simple methods to process text-annotations and map them to ontologies to demonstrate that such automatic mapping enables integration of gene expression and tissue microarray datasets.

Currently the only way to query the processed annotations is via SQL queries. Performing complicated SQL queries is not always possible for all end-users and the ontology hierarchies and the mapped annotations should drive specialized query-interfaces [9]. One possible approach is to process the user's query text using the same indexing method that mapped the annotations of the datasets and retrieve those datasets that have the largest intersection with the concepts identified in the processed query. In future we will develop interfaces to search these processed annotations as part of our work at the National Center for Biomedical Ontology, to create resources and methods to (help biomedical investigators) store, view, and compare annotations of biomedical research data.

As we all know, genomic scale data is increasing in volume and diversity. Currently, little attention is being paid to the problem of developing methods to integrate complementary data types such as gene expression microarrays and tissue microarrays based on their annotations [3,5].

We believe it is possible to perform ontology based indexing of free-text annotations of high throughput datasets in public databases to enable such integration. If the annotations of multiple databases – such as that of GEO, TMAD, SMD, PharmGKB – are processed in this manner, it will enable users to perform integrated analyses of multiple, high throughput, genomic scale dataset s[5]. In order to demonstrate the feasibility as well as utility of ontology based indexing, we have developed a prototype system to annotate elements from public biomedical resources and enable users to search the indexed resources in an ontology driven manner.

## Conclusion

We have demonstrated that we can effectively map the text-annotations of microarray datasets in GEO as well as annotations of tissue microarrays in TMAD to concepts from vocabularies in the UMLS. Our results show that we can map disease names and disease related concepts with high precision and recall.

We demonstrate how such mapping to concept hierarchies offers the ability to identify corresponding datasets across different repositories for integrative analyses. We identified 23 candidate datasets for further study. We have implemented the mapping functionality as a PERL module named UMLS-Query, which is available with documentation at [16].

We have described a prototype implementation of an ontology-based annotation and indexing system. The system processes text metadata of gene-expression data sets, descriptions of radiology images, clinical-trial reports, as well as abstracts of PubMed articles to annotate them automatically with concepts from appropriate ontologies. The system enables researchers to locate relevant biological data sets for integrative analyses.

## Competing interests

The authors declare that they have no competing interests.

## Authors' contributions

NHS conceived of the project and performed the proof of concept implementation on GEO and TMAD. CM implemented the prototype at [12], APC performed the evaluations, and RC maintained the local GEO installation. AJB and MAM contributed to the manuscript, provided critical feedback, supervision and direction to the project. All authors approved the final manuscript.
